# DRAWID: user-friendly java software for chromosome measurements and idiogram drawing

**DOI:** 10.3897/CompCytogen.v11i4.20830

**Published:** 2017-11-21

**Authors:** Ilya Kirov, Ludmila Khrustaleva, Katrijn Van Laere, Alexander Soloviev, Sofie Meeus, Dmitry Romanov, Igor Fesenko

**Affiliations:** 1 Shemyakin-Ovchinnikov Institute of Bioorganic Chemistry, Russian Academy of Sciences, Laboratory of proteomics, Miklukho-Maklaya, 16/10,117997, Moscow, Russia; 2 Russian State Agrarian University-MTAA, Timiryazevskay str. 49, 127550 Moscow, Russia; 3 Institute for Agricultural and Fisheries Research (ILVO), Plant Sciences Unit, Applied Genetics and Breeding, Caritasstraat 21, 9090 Melle, Belgium

**Keywords:** Karyotyping, idiogram, chromosome software, plant cytogenetics

## Abstract

An idiogram construction following chromosome measurements is a versatile tool for cytological, cytogenetic and phylogenetic studies. The information on chromosome length, centromere index and position of cytogenetic landmarks along with modern techniques (e.g. genomic and fluorescence in situ hybridization, banding, chromosome painting) can help to shed light on genome constitution, chromosome rearrangements and evolution. While idiogram construction is a routine task there are only few freely available programs that can perform chromosome measurements and no software for simultaneous measuring of chromosome parameters, chromosomal landmark and FISH signal positions and idiogram construction. To fill this gap, we developed DRAWID (DRAWing IDiogram), java-based cross-platforming program for chromosome analysis and idiogram construction. DRAWID has number of advantages including a user-friendly interactive interface, possibility for simultaneous chromosome and FISH/GISH/banding signal measurement and idiogram drawing as well as number of useful functions facilitating the procedure of chromosome analysis. The output of the program is Microsoft XL table and publish-ready idiogram picture. DRAWID and the manual for its use are freely available on the website at: http://www.drawid.xyz

## Introduction

Chromosome number, morphology and organization are important parameters for comparative cytogenetic and phylogenetic studies (Mandáková and Lysak 2008; Peruzzi et al. 2009; Cheng et al. 2013; Kirov et al. 2014; Divashuk et al. 2014; Bolsheva et al. 2015; Astuti et al. 2017). Differences in chromosome morphology between individual species are the result of inter- and intra-chromosomal rearrangements which are major forces of evolution and speciation (Rieseberg 2001; De Storme and Mason 2014; Mandáková et al. 2015). Knowledge about chromosome rearrangements and basic chromosome characteristics (e.g. centromere index, arm ratio, relative length, chromosomal asymmetry) can also be useful for the integration of physical and genetic maps, the study of speciation and evolution and for tracing desirable traits during plant breeding processes (Peruzzi and Eroğlu 2013; Budylin et al. 2014; Laskowska et al. 2015; Astuti et al. 2017). Modern molecular cytogenetic techniques, e.g. genomic in situ hybridization (GISH), further help to shed light on karyotype constitution and chromosomal rearrangements (Van Laere et al. 2010; Laskowska et al. 2015). Chromosome number and structure are typically schematically represented in an idiogram, showing chromosome length, centromere index and chromosome arm ratio. Measurements on at least 5 metaphase plates are used to build an idiogram. Moreover, to determine the correct chromosome order and homologous pairs, additional chromosomal markers, such as banding patterns and FISH signals, are often required. For plant species like *Arabidopsis thaliana* (Linnaeus, 1753) Heynhold, 1842 , wheat and maize (*Triticum aestivum* Linnaeus, 1753 and *Zea mays* Linnaeus, 1753, respectively), a well-defined karyotype and idiogram have been published, facilitating cytogenetic studies of their genomes (Gill et al. 1991; Fransz et al. 1998; Sadder and Weber 2001; Badaeva et al. 2007). However, for most plant genera this information is lacking and chromosome measurements are needed to build a karyotype. In addition, cytology-based ecological studies of genome variability require measurements of chromosomes from a large number of individuals.

To accelerate karyotype studies in plants only few software programs are available, including MicroMeasure (Reeves and Tear 2000), IdeoKar (Mirzaghaderi and Marzangi 2015) and KaryoType (Altınordu et al. 2016). These programs allow measurement of chromosome parameters such as centromere index, arm length and ratio, asymmetry index, etc. However, none of these programs is able to simultaneously measure chromosome parameters and chromosomal landmark positions (e.g. band, FISH and GISH signals), allowing idiogram construction.

Here, we present the DRAWID (**DRAW**ing **ID**iogram) – program for chromosome analysis and idiogram construction. DRAWID is a user-friendly and freely available (under GNU General Public License) java-based software program that facilitates basic as well as ISH-based karyotype analysis. DRAWID is equipped with an intuitive graphical user interface. Input files for DRAWID are image files (JPEG and PNG) or data tables generated by DRAWID itself. Output of the program are Microsoft XL (2010) tables, containing measurement details (centromere index, arm ratio, relative and absolute length of chromosome and chromosome arms, signal and band positions and size (if available), and DRAWID-built idiogram pictures. The idiogram parameters can be easily adjusted to prepare a high-quality image suitable for publication. In addition, to facilitate high-throughput karyotyping the program enables to collect data from different metaphases, and construct an average idiogram with error bars representing the standard deviation for chromosome length and centromere position.

We designed a web page on the website of the Russian State Agrarian University-MTAA (Department of plant genetics, biotechnology and breeding) from which DRAWID v0.26 can be downloaded, together with the manual for its use and possibility for bug reports (http://www.drawid.xyz).

## Material and methods

### Software development

The original code of the program was written in Java 8 using IntelliJ IDEA as the integrated development environment and is compatible with any Java-enable system with a runtime level of ≥1.7. Microsoft Excel version 10.0 or higher is required.

### Abbreviations

**FISH** Fluorescent in situ hybridization

**PCR** Polymerase chain reaction

**GISH** Genomic in situ hybridization

### FISH and GISH

For cytogenetic experiments chromosomes were prepared using the SteamDrop method (Kirov et al. 2014). To visualise 5S and 45S rDNA in *Allium fistulosum* Linnaeus 1753, the plasmids pSct7 (Lawrence and Appels 1986) and pTa71 (Gerlach and Bedbrook 1979) were used. FISH was performed as described in Kirov et al. (2016). Biotin and digoxigenin labeled probes were detected by Streptavidin-Cy3 (Sigma-Aldrich, USA) and anti-digoxigenin-FITC (Roche, Germany), respectively.

GISH on × *Festulolium* Ascherson & Graebner, 1902 hybrids was performed as described in Van Laere et al (2010). *Lolium perenne* Linnaeus, 1753 was used as probe DNA, labelled with Digoxigenin, while *Festuca pratensis* Hudson, 1762 was used as block DNA.

### Microscopy and image analysis

Images were taken by a Zeiss AxioImager M1 fluorescence microscope (400× and 1000× magnification) equipped with an AxioCam MRm camera and ZEN software (Zeiss, Zaventem, Belgium).

## Results and discussion

### Implementation

**Figure 1.**
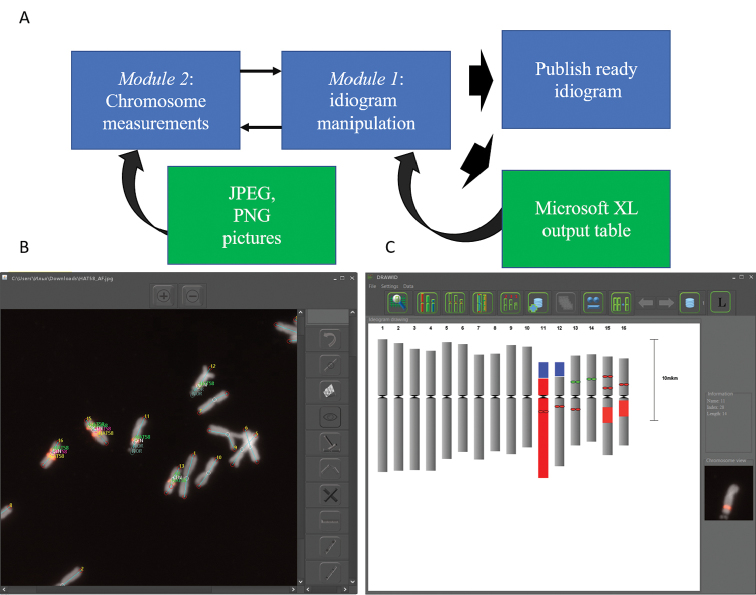
Structure (**A**) and main windows of DRAWID (**B, C**). **B** Interface of chromosome measurement window, containing useful tools for chromosome and FISH/GISH signal measurements. The photograph in this panel shows the result of a FISH experiment on chromosomes of *Allium fistulosum* with biotin-labeled 5S rDNA and HAT58 repeat (Kirov et al. 2017). Lines and signatures show the path of chromosome and signal measuring **C** Interface for idiogram manipulation. This panel shows the idiogram of *A. fistulosum* constructed based on chromosome measurements and FISH (5S rDNA and HAT58 tandem repeat) signal positions in panel **B** Buttons at the top of the panel are used for chromosome and centromere color changing, display legend, chromosome order correction and idiogram storage manipulation. When a chromosome in the constructed idiogram is selected (entire chromosome 11 highlighted in red the image of the selected chromosome along with the parameters of its measurement appear on the screen (on the right of the panel **C**).

DRAWID contains two main modules: (1) idiogram manipulations and (2) chromosome measurements (Figure 1A). DRAWID can draw an idiogram using data from the second module as well as from DRAWID generated Microsoft XL output tables. The first module provides an interface for idiogram manipulation, storage, analysis as well as for data representation. Chromosome order, name, and color and centromere color can be changed using the top menu and the pop-up menu after chromosome selection. Once idiogram adjustments are performed, the idiogram can be added to the DRAWID storage to use it later again. From this window high quality pictures can be saved in PNG format. Data from DRAWID can be saved as XL Microsoft files (.xlsx). The excel data files contain two sheets in total, one sheet with chromosome parameters such as chromosome length, centromere index (short arm length × 100 / total chromosome length), relative chromosome length, short arm and long arm lengths, and one metadata sheet containing information about signal and band positions and size. Building an idiogram for cytogenetically uncharacterized species requires an average idiogram based on measurements of several metaphases. To facilitate this process we implemented a function to build an average karyotype from multiple datasets stored in the DRAWID storage. After application of this function measurements of chromosomes with the same names will be used to calculate means for centromere index, whole chromosome length, short and long arm lengths and standard deviation. An average idiogram is plotted with error bars representing the standard deviation. This idiogram can again be exported and saved either as an image file or as a table. To build idiograms for haploids we inserted a function to merge neighboring (chromosome ordering is just performed by length) chromosomes depending on the ploidy level.

The second module (Figure 1A, B) allows chromosome measurements as well as chromosomal landmarking and includes a number of useful tools to simplify the measurement process. Chromosome and landmark names appear on the picture once the measurement is completed. FISH signal positions can be drawn on the idiogram. Correct positioning of the signals on the idiogram is becoming annoying when many probes and/or loci are involved. Therefore, DRAWID is equipped by a set of functions to correctly position all the signals on the idiogram and to easlily change their colors and names using the icons in the top panel menu. Chromosome name changes in the idiogram are automatically and immediately synchronized with the measurements on the picture. In addition, the visualized chromosome on the right side of the panel helps to identify the chromosomes and to link the idogram to the original picture. Sometimes, chromosomes have fragile sites and as a consequence, different fragments of these chromosomes can be positioned on different locations in the metaphase. To virtually join two or more fragments of the same chromosome and build an idiogram, we also implemented a specific function.

All measurements can be scaled by measuring the scale bar in the picture and using the scale bar button. As some errors can occur during measurements, DRAWID has several functions to remove measurements of certain chromosomes. Some frequently used functions such as marking a coordinate as a centromere or FISH signal/band, finishing chromosome measurements have both a hotkey and an icon.

The DRAWID program has a dedicated web page (http://www.drawid.xyz) on the website of the Department of plant genetics, biotechnology and breeding of Russian State Agrarian University – MTAA. All the described functions and some other functions are explained there in detail in the manual. In addition, version history and information about reported and solved bugs are published here and will be updated on a regular basis.

### Validation

**Example 1. Basic karyotyping and averaged idiogram**

**Figure 2.**
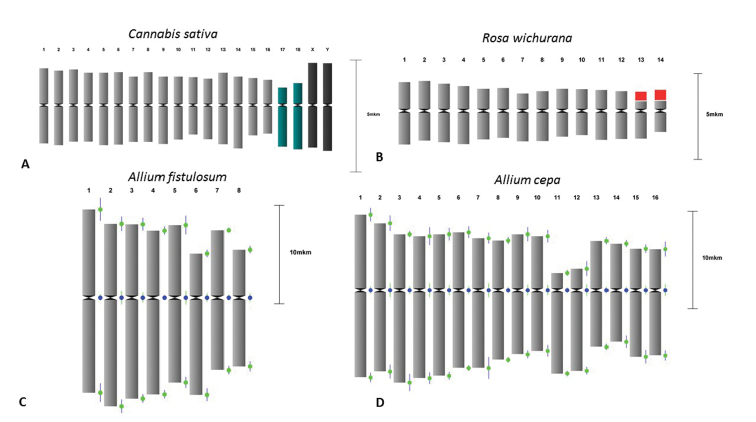
Examples of basic karyotype measurements and idiogram constuction by DRAWID. **A** Chromosomes of *C. sativa* (2*n* = 20); sex (black color) and NOR-bearing (green color) chromosomes are highlighted **B** Idiogram of *Rosa wichurana*; satellites on chromosomes 13 and 14 are colored in red **C** Idiogram of *Allium fistulosum* after measurement of the chromosomes, and application of the function “reduce karyo” merging homologuous chromosomes to obtain a monoploid idiogram, standard deviation bars are shown **D** Idiogram of *Allium cepa* constructed after measurements of 3 metaphases and application of the “get average karyo” function to obtain the average idiogram, standard deviation bars are shown.

In order to assess DRAWID for karyotyping of individual metaphases (Figure 2A, B) as well as of a set of metaphases (Figure 2C, D), we used previously published data from karyotyping of *Cannabis sativa* Linnaeus, 1753 (Divashuk et al. 2014), *Rosa wichurana* Crépin, 1888 (Kirov et al. 2016), *Allium cepa* Linnaeus, 1753 and *A. fistulosum* (de Vries and Jongerius 1988, Kirov et al. 2017). All tested species are diploid, 2*n* = 2*x* = 20 (*Cannabis sativa*), 2*n* = 2*x* = 14 (*Rosa wichurana*), 2*n* = 2*x* = 16 (*Allium cepa* and *A. fistulosum*). Idiograms for these species constructed by DRAWID are presented in Figure 2. Our results fully coincided with the data of karyotyping published earlier.

The idiogram can also represent a monoploid chromosome set. For this, DRAWID has a function to convert a diploid or polyploid idiogram into a monoploid one and calculate the mean chromosome index and arm length of homologuous chromosomes. A new idiogram can then be drawn with indication of standard deviation bars. To demonstrate this, measurements of *Allium fistulosum* metaphase and idiogram construction using the function ‘reduce karyo’ was performed in DRAWID (Figure 2C).

In another example, three metaphases of *Allium cepa* were measured, data were collected into the storage container and an average idiogram was obtained using the “average karyo” function (Figure 2D). Standard deviations of chromosome arm lenghts and centromere indices are presented which provide useful information for the estimation of chromosome parameter variability and comparative analysis.

**Example 2. FISH based idiogram**

**Figure 3.**
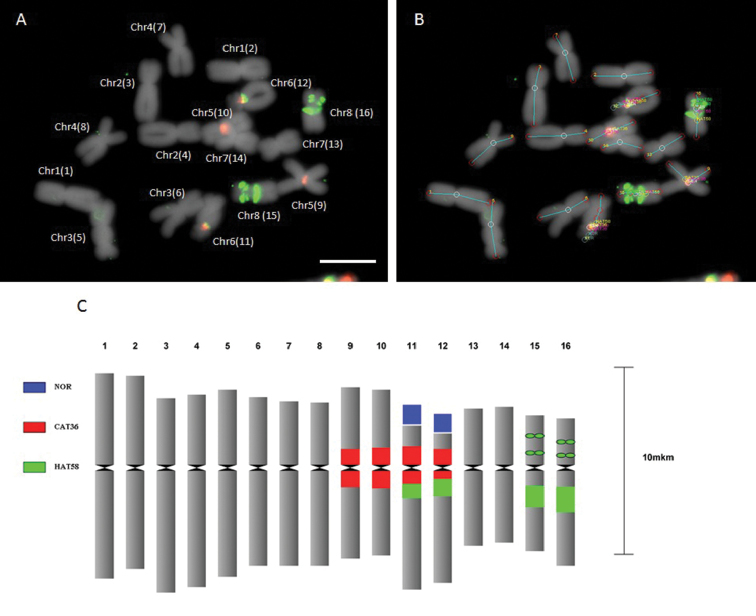
**A** Metaphase chromosomes of *A. fistulosum* after FISH with HAT58 (green signal) and CAT36 (red signals) repeats. Numbers in brackets correspond to the numbers on the idiogram (**C**) **B** The same picture as in **A** but after measurements by DRAWID **C** Idiogram obtained from the measurements in **A** and **B** Scale bar – 10 µm.

One major added value of DRAWID compared to other software is that it allows to measure FISH signals and indicate them on the idiogram. Figure 3 shows an idiogram construction of an *A. cepa* metaphase with HAT58 and CAT36 signals. The position of the signals and size (in case of bands) were calculated. Based on these measurements, the idiogram was build by DRAWID, using the scale bar for calibration, and signal positions of HAT58 and CAT36 rDNA probes were indicated.

**Example 3. Idiogram after GISH experiments**

**Figure 4.**
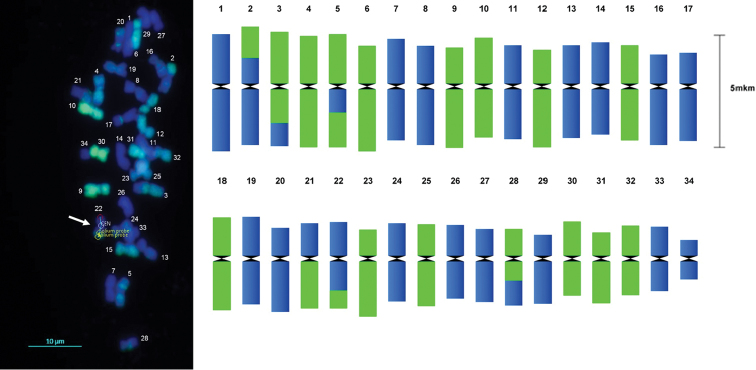
Idiogram (right) obtained from metaphase measurements of an F2 × *Festulolium* hybrid (2*n* = 34) after GISH analysis (left) with *Lolium perenne* genomic DNA labeled as a probe (Dig; green pseudocolor) and *Festuca pratensis* used as block DNA, counterstained with DAPI (blue pseudocolor). DRAWID measurements are shown for the recombinant chromosome 22 (arrow, CEN = centromere). Chromosome numbering is according to chromosome length starting with the largest chromosome.

GISH is a commonly used tool to study genome composition after interspecific crosses. A correctly drawn idiogram with indication of recombination points is important for result interpretation. We tested DRAWID to build idiograms from F2 hybrids between species of *Lolium* Linnaeus, 1753 and *Festuca* Linnaeus, 1753, having a complex genomic constitution with several recombination points. Using DRAWID, chromosome number, chromosome morphology and GISH signals were determined. On the idiogram, parental composition and sites of recombination are clearly visible (Figure 4).

## Conclusion

Modern molecular cytogenetics and cytology requires easy-to-use software for measurements of chromosomes. Exciting advances in FISH technology significantly expanded the boundaries of cytogenetics. FISH is a frequently used tool for plant chromosome identification, monitoring of allien DNA in hybrids, evolutionary studies, physical map construction etc. Here, we present a new software called DRAWID, containing a number of useful functions to make processing of chromosome measurements, FISH signal mapping and preparing of publishable idiograms as easy as possible. DRAWID has a number of advantages compared to previously published programs: 1) simultaneous drawing of idiogram, FISH/GISH/banding signals and measuring; 2) easy adjustment of idiogram color, chromosome position and names, 3) possibility to build average idiograms (with error bars) from collections of single metaphase idiograms. In the future, new functions can be added to further simplify the process of cytogenetic image analysis and idiogram drawing. All updates will be immediately available for the scientific community.

### Authors’ contributions

IK, LK, KVL wrote the paper and designed the experiments. DR, AS, SM, IF tested and debugged the program. IK designed the webpage.

## References

[B1] Altınordu F Peruzzi L Yu Y He X (2016) A tool for the analysis of chromosomes: KaryoType. Taxon 65(3): 586–592. https://doi.org/10.12705/653.9

[B2] Astuti G Roma-Marzio F Peruzzi L (2017) Traditional karyomorphological studies: can they still provide a solid basis in plant systematics? Flora Mediterranea 27: 91–98.

[B3] Bolsheva NL Zelenin AV Nosova IV Amosova AV Samatadze TE Yurkevich OY Melnikova NV Zelenina DA Volkov AA Muravenko OV (2015) The diversity of karyotypes and genomes within section Syllinum of the genus *Linum* (Linaceae) revealed by molecular cytogenetic markers and RAPD analysis. PLoS ONE 10(4): e0122015. https://doi.org/10.1371/journal.pone.0122015 10.1371/journal.pone.0122015 PMC4383504 25835524

[B4] Badaeva ED Dedkova OS Gay G Pukhalskyi VA Zelenin AV Bernard S Bernard M (2007) Chromosomal rearrangements in wheat: their types and distribution. Genome 50(10): 907–926. https://doi.org/10.1139/G07-072 18059554 10.1139/g07-072

[B5] Budylin M Kan L Romanov V Khrustaleva L (2014) GISH study of advanced generation of the interspecific hybrids between *Allium cepa* L. and *Allium fistulosum* L. with relative resistance to downy mildew. Russian Journal of Genetics 50(4): 387–394. https://doi.org/10.1134/S1022795414040036 25715446

[B6] Cheng F Mandáková T Wu J Xie Q Lysak MA Wang X (2013) Deciphering the diploid ancestral genome of the mesohexaploid *Brassica rapa*. The Plant Cell 25(5): 1541–1554. https://doi.org/10.1105/tpc.113.110486 23653472 10.1105/tpc.113.110486 PMC3694691

[B7] Divashuk MG Alexandrov OS Razumova OV Kirov IV Karlov GI (2014) . Molecular cytogenetic characterization of the dioecious *Cannabis sativa* with an XY chromosome sex determination system. PLoS One 9(1): e85118. https://doi.org/10.1371/journal.pone.0085118 10.1371/journal.pone.0085118 PMC3897423 24465491

[B8] De Storme N Mason A (2014) Plant speciation through chromosome instability and ploidy change: cellular mechanisms, molecular factors and evolutionary relevance. Current Plant Biology 1: 10–33. https://doi.org/10.1016/j.cpb.2014.09.002

[B9] De Vries JN MC Jongerius (1988) Interstitial C-bands on the chromosomes of Allium-species from section Cepa. Proceeding of 4th Eucarpia Allium Symposium, 71–78.

[B10] Fransz P Armstrong S Alonso‐blanco C Fischer TC Torres‐ruiz RA Jones G (1998) Cytogenetics for the model system Arabidopsis thaliana. The Plant Journal 13(6): 867–876. https://doi.org/10.1046/j.1365-313X.1998.00086.x 9681023 10.1046/j.1365-313x.1998.00086.x

[B11] Gerlach WL Bedbrook JR (1979) Cloning and characterization of ribosomal RNA genes from wheat and barley. Nucleic Acids Research 7: 1869–1885 https://doi.org/10.1093/nar/7.7.1869 537913 10.1093/nar/7.7.1869 PMC342353

[B12] Gill BS Friebe B Endo TR (1991) Standard karyotype and nomenclature system for description of chromosome bands and structural aberrations in wheat (*Triticum aestivum*). Genome 34(5): 830–839. https://doi.org/10.1139/g91-128

[B13] Heslop-Harrison JS Schwarzacher T Anamthawat-Jénsson K Leitch AR Shi M Leitch IJ (1991) In-situ hybridization with automated chromosome denaturation. Technique 3: 109–116.

[B14] Kirov I Van Laere K De Riek J De Keyse E Van Roy N Khrustaleva L (2014) Anchoring linkage groups of the *Rosa* genetic map to physical chromosomes with Tyramide-FISH and EST-SNP markers. PloS One 9(4): e95793. https://doi.org/10.1371/journal.pone.0095793 10.1371/journal.pone.0095793 PMC3995938 24755945

[B15] Kirov IV Van Laere K Van Roy N Khrustaleva LI (2016) Towards a FISH-based karyotype of *Rosa* L.(Rosaceae). Comparative Cytogenetics 10(4): 543. https://doi.org/10.3897/compcytogen.v10i4.9536 10.3897/CompCytogen.v10i4.9536 PMC5240508 28123677

[B16] Kirov I Divashuk M Van Laere K Soloviev A Khrustaleva L (2014) An easy “SteamDrop” method for high quality plant chromosome preparation. Molecular Cytogenetics 7(1): 21. https://doi.org/10.1186/1755-8166-7-21 10.1186/1755-8166-7-21 PMC3995958 24602284

[B17] Laskowska D Berbeć A Van Laere K Kirov I Czubacka A Trojak-Goluch A (2015) Cytology and fertility of amphidiploid hybrids between *Nicotiana wuttkei* Clarkson et Symon and *N. tabacum* L. Euphytica 206(3): 597–608. https://doi.org/10.1007/s10681-015-1459-3

[B18] Lawrence GJ Appels R (1986) Mapping the nucleolus organizer region, seed protein loci and isozyme loci on chromosome 1R in rye. TAG Theoretical and Applied Genetics 71(5): 742–749. https://doi.org/10.1007/BF00263273 24247611 10.1007/BF00263273

[B19] Mandáková T Lysak MA (2008) Chromosomal phylogeny and karyotype evolution in *x* = 7 crucifer species (Brassicaceae). The Plant Cell 20(10): 2559–2570. https://doi.org/10.1105/tpc.108.062166 18836039 10.1105/tpc.108.062166 PMC2590746

[B20] Mandáková T Singh V Krämer U Lysak MA (2015) Genome structure of the heavy metal hyperaccumulator *Noccaea caerulescens* and its stability on metalliferous and nonmetalliferous soils. Plant Physiology 169(1): 674–689. https://doi.org/10.1104/pp.15.00619 26195571 10.1104/pp.15.00619 PMC4577401

[B21] Mirzaghaderi G Marzangi K (2015) IdeoKar: an ideogram constructing and karyotype analyzing software. Caryologia 68(1): 31–35. https://doi.org/10.1080/00087114.2014.998526

[B22] Peruzzi L Leitch IJ Caparelli KF (2009) Chromosome diversity and evolution in Liliaceae. Annals of Botany 103(3): 459–475. https://doi.org/10.1093/aob/mcn230 19033282 10.1093/aob/mcn230 PMC2707325

[B23] Peruzzi L Eroğlu HE (2013) Karyotype asymmetry: again, how to measure and what to measure? Comparative cytogenetics 7(1): 1–9. https://doi.org/10.3897/compcytogen.v7i1.4431 10.3897/CompCytogen.v7i1.4431 PMC3833747 24260685

[B24] Reeves A Tear J (2000) MicroMeasure for Windows. Version 3.3. http://www.colostate.edu/Depts/Biology/MicroMeasure [Accessed 25 July 2017]

[B25] Rieseberg LH (2001) Chromosomal rearrangements and speciation. Trends in Ecology & Evolution 16(7): 351–358. https://doi.org/10.1016/S0169-5347(01)02187-5 11403867 10.1016/s0169-5347(01)02187-5

[B26] Sadder MT Weber G (2001) Karyotype of maize (Zea mays L.) mitotic metaphase chromosomes as revealed by fluorescencein situ hybridization (FISH) with cytogenetic DNA markers. Plant Molecular Biology Reporter 19(2): 117–123. https://doi.org/10.1007/BF02772153

[B27] Van Laere K Khrustaleva L Van Huylenbroeck J Van Bockstaele E (2010) Application of GISH to characterize woody ornamental hybrids with small genomes and chromosomes. Plant Breeding 129(4): 442–447.

